# Sex Disparities in *Staphylococcus aureus* Bacteremia Mortality

**DOI:** 10.1001/jamanetworkopen.2024.41502

**Published:** 2024-10-30

**Authors:** Bethan L. Carter, Jonathan Underwood

**Affiliations:** 1 Division of Infection and Immunity, School of Medicine, Cardiff University, Cardiff, United Kingdom; 2 Department of Infectious Diseases, Cardiff and Vale University Health Board, Cardiff, United Kingdom

## Abstract

This cohort study examines sex differences in 30- and 90-day mortality among patients with *Staphylococcus aureus* bacteremia in Wales, United Kingdom.

## Introduction

*Staphylococcus aureus* bacteremia (SAB) poses a significant global health threat, with 30-day mortality rates estimated at approximately 20%.^1^ A recent systematic review and meta-analysis reported female sex was associated with increased SAB mortality, with female patients at 18% increased odds of death compared with male patients.^2^ Attempting to corroborate this apparent sex disparity, we reanalyzed data from our own nationwide study of mortality among adults with SAB in Wales, UK.^3^

## Methods

This retrospective cohort study was conducted within the Secure Anonymized Information Linkage Databank (SAIL), containing anonymized population-scale electronic health record data for Wales, using previously described methods.^3^ Proposals using SAIL data require review by an independent Information Governance Review Panel. This work was approved under proposal 0923 and followed the Strengthening the Reporting of Observational Studies in Epidemiology (STROBE) reporting guideline. Informed patient consent was not required due to the use of anonymized individual-level data sources held within the Trusted Research Environment provided by the SAIL Databank at Swansea University, Swansea, UK. We included adults with SAB between April 1, 2010, and March 31, 2022. Demographic characteristics were compared by sex using Wilcoxon rank sum or Pearson χ^2^ tests, as appropriate. All-cause mortality within 30 and 90 days were compared using logistic regression with results adjusted for baseline covariates, including age, hospital or community onset, methicillin sensitivity, Charlson comorbidity index (CCI) score, Welsh Index of Multiple Deprivation (a measure of relative deprivation of an area [in this case, the postcode of the participant] based on 8 components, including income, employment, and education),^4^ and electronic frailty index (a cumulative deficit model that assigns a frailty score to an individual calculated using 36 variables from electronic primary care data including symptoms, signs, diseases, disabilities, and laboratory results).^5^ Competing risk regression, a proportional subdistribution hazards regression model (with death from a nonsepsis cause as a competing risk) was used to determine the association of female sex with 30-day sepsis-specific mortality adjusting for the same confounders. *P* values were 2-sided, and *P* < .05 was considered statistically significant for all statistical tests. There was no imputation for missing data. Analyses were conducted using R software version 4.1.3 (R Project for Statistical Computing from May 5 to August 23, 2024.

## Results

A total of 7515 adults with SAB (median [IQR] age, 70 [55-81] years) were identified, including 4755 male patients (63%) and 2760 female patients (37%). Complete demographics can be found in Table 1. Overall mortality at 30 days after SAB was 2057 deaths (27%), including 1262 among male patients (27%) and 795 among female patients (29%). Overall mortality at 90-day post-SAB mortality was 2712 (36%), including 1690 among male patients (36%) and 1022 among female patients (37%).

**Table 1.** Baseline Demographics of SAB Patients Stratified by Sex

Variable	Sex, No. (%)		*P* value^a^
	Male (n = 4755)	Female (n = 2760)	
Age, median (IQR), y	70 (55-80)	71 (55-82)	.07
Frailty rating			
Fit	2009 (42)	1018 (37)	<.001
Mild	1428 (30)	865 (31)	
Moderate	928 (20)	624 (23)	
Severe	390 (8)	253 (9)	
Charlson comorbidity index, median (IQR)	10 (0-22)	8 (0-18)	<.001
Welsh Index of Multiple Deprivation			
5 (least deprived)	708 (16)	440 (17)	.43
4	757 (17)	433 (17)	
3	898 (20)	544 (21)	
2	1017 (23)	549 (21)	
1 (most deprived)	1115 (25)	647 (25)	
Missing	260	147	NA
Attribution			
Community	2115 (44)	1130 (41)	.003
Hospital	2640 (56)	1630 (59)	
MRSA	646 (14)	311 (11)	.004
CRP, mg/dL			
Under 100	788 (19)	507 (21)	<.001
10-20	1274 (30)	558 (23)	
20-30	1099 (26)	623 (26)	
30-40	759 (18)	466 (19)	
≥40	304 (7)	283 (12)	
Missing	531	323	NA
Peak CRP, median (IQR), mg/L	205 (122-301)	225 (118-323)	<.001
30-d mortality	1262 (27)	795 (29)	.03
90-d mortality	1690 (36)	1022 (37)	.20

Abbreviations: CRP, C-reactive protein; MRSA, methicillin-resistant *Staphylococcus aureus*; NA, not applicable.

SI conversion factor: To convert C-reactive protein to milligrams per liter, multiple by 10.

^a^ Wilcoxon rank sum test; Pearson χ^2^ test.

In unadjusted models, female sex was associated with greater all-cause SAB mortality at 30 days (odds ratio [OR], 1.12; 95% CI, 1.01-1.24; *P* = .03) but not at 90 days (OR, 1.07; 95% CI, 0.97-1.18; *P* = .20) (Table 2). However, in adjusted models, female sex was associated with both 30-day (OR, 1.19; 95% CI, 1.06-1.34; *P* = .003) and 90-day (OR, 1.15; 95% CI, 1.03-1.29; *P* = .02) mortality (Table 2). Using an adjusted competing risks regression model for 30-day mortality, female sex was associated with SAB deaths due to sepsis (hazard ratio, 1.21; 95% CI, 1.02-1.44; *P* = .03) but not with other causes (hazard ratio, 1.10; 95% CI, 0.99-1.23; *P* = .08).

**Table 2.** Logistic Regression Models for 30- and 90-Day All-Cause Mortality Following *Staphylococcus aureus* Bacteremia

Sex	Unadjusted				Adjusted			
	No.	Deaths	OR (95% CI)	*P* value	No.	Deaths	OR (95% CI)^a^	*P* value
**30-d mortality**								
No.	7515	2057	NA	NA	7108	1934	NA	NA
Male	4755	1262	1 [Reference]	.03	4495	1189	1 [Reference]	.003
Female	2760	795	1.12 (1.01-1.24)		2613	745	1.19 (1.06-1.34)	
**90-d mortality**								
No.	7515	2712	NA	NA	7108	2560	NA	NA
Male	4755	1690	1 [Reference]	.20	4495	1598	1 [Reference]	.02
Female	2760	1022	1.07 (0.97-1.18)		2613	962	1.15 (1.03-1.29)	

Abbreviations: NA, not applicable; OR, odds ratio.

^a^ Adjusted for methicillin-resistant *Staphylococcus aureus *and methicillin-susceptible *Staphylococcus aureus*, age, Welsh Index of Multiple Deprivation, Charlson comorbidity index, attribution, and frailty.

## Discussion

The findings from our cohort study reaffirm the observed increased mortality among female patients with SAB. Furthermore, our data suggest that sepsis-related mortality in particular was the underlying cause of this disparity.

Greater sepsis-related mortality and higher C-reactive protein in females may be indicative of sex-based differences in immune response to SAB, potentially influenced by X-chromosome genetic polymorphisms and variations in Toll-like receptor expression and signaling.^6^ However, this hypothesis remains speculative, as pathogen-specific immune data from female patients are still lacking. Alternative explanations for our findings may include sex-based differences in source of SAB, health care–seeking behaviors, or health care delivery.

This study has some limitations, including the lack of sex-specific data on the initial source of SAB. Additional limitations are described in our previous study.^3^ However, a large, unselected cohort, coupled with complete SAIL data, historical microbiological records, and thorough adjustment for confounders strengthen the reliability of our results.

Further research is necessary to investigate underlying pathophysiological, social, and health care–related factors that underpin sex-related mortality differences in SAB. Addressing these disparities could lead to more targeted therapies, improved survival rates, and more equitable health care outcomes for both sexes.

## Notes

Supplement.
Data Sharing Statement
