# Supplementary material for: Sex Disparities in Staphylococcus aureus Bacteremia Mortality
Source: JAMA Netw Open. 2024 Oct 30;7(10):e2441502. doi: 10.1001/jamanetworkopen.2024.41502 (PMC11525600; doi:10.1001/jamanetworkopen.2024.41502)
Supplement: Supplement. — Data Sharing Statement [file jamanetwopen-e2441502-s001.pdf]

Data Sharing Statement

Carter. Sex Disparities in Staphylococcus aureus Bacteremia Mortality. *JAMA Netw Open*. Published October 30, 2024. doi:10.1001/jamanetworkopen.2024.41502

Data
Data available: Yes

Additional Information
The data used in this study are available in the SAIL Databank at Swansea University, Swansea, UK, but as restrictions apply, they are not publicly available. All proposals to use SAIL data are subject to review by an independent Information Governance Review Panel (IGRP). Before any data can be accessed, approval must be given by the IGRP. The IGRP carefully considers each project to ensure the proper and appropriate use of SAIL data. When access has been granted, it is gained through a privacy-protecting trusted research environment (TRE) and remote access system referred to as the SAIL Gateway. SAIL has established an application process to be followed by anyone who would like to access data via SAIL at https://www.saildatabank.com/application-process. The R code used for data analysis is available at written request to the corresponding author.
